# Supplementary material for: Radiological Assessment in Idiopathic Pulmonary Fibrosis (IPF) Patients According to MUC5B Polymorphism
Source: Int J Mol Sci. 2022 Dec 14;23(24):15890. doi: 10.3390/ijms232415890 (PMC9784960; doi:10.3390/ijms232415890)
Supplement: Supplementary file 1 [file ijms-23-15890-s001.zip › ijms-2023935-Supplementary.pdf]

International Journal of
Molecular Sciences

MDPI

Supplementary Materials

Radiological Assessment in Idiopathic Pulmonary Fibrosis (IPF) Patients According to MUC5B Polymorphism

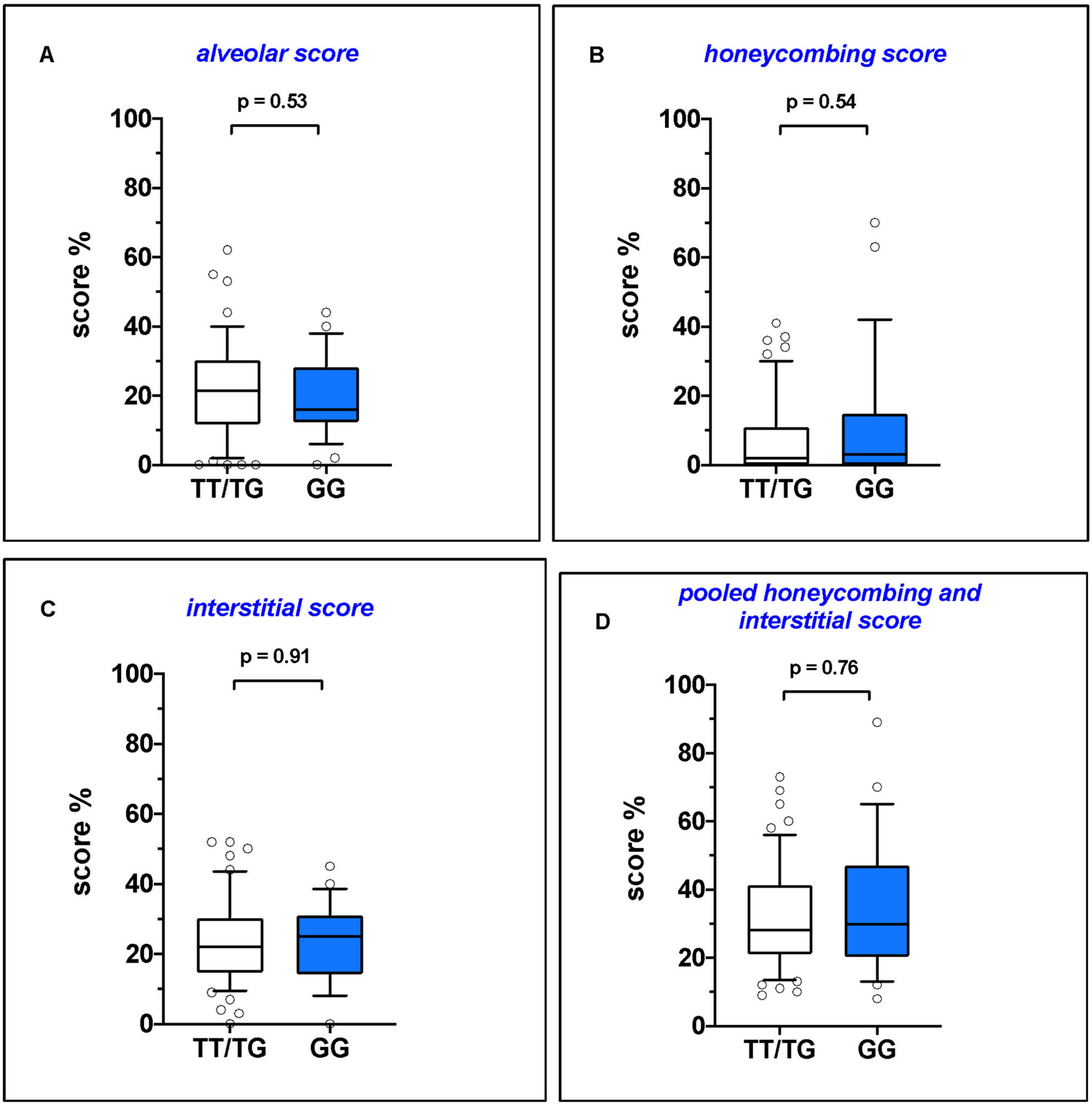

Figure S1. Radiological scores at treatment initiation (HRCT1) of the study population cathegorized in TT/TG genotype or GG genotype. Values of alveolar score (Panel A), honeycombing score (panel B), interstitial score (panel C) and pooled honeycombing and interstitial score (Panel D) at treatment initiation (HRCT1) in TT/TG genotype patients (TT/TG) and GG genotype patients (GG). Horizontal bars represent median values; bottom and top of each box plot 25th and 75th, brackets 10th and 90th percentiles, while circles represent outliers. White boxes indicate TT/TG genotype patients and blue boxes GG genotype patients.

Table S1. Patient's demographics and clinical characteristics of the entire study population, and cathegorized in TT/TG genotype or GG gentotype.

	Entire Population (n =78)	TT/TG genotype (n =54)	GG genotype (n =24)	p Value
**Male – n (%)**	64 (82)	44 (82)	20 (83)	0.84
**Female – n (%)**	14 (18)	10 (18)	4 (17)	
**Age at diagnosis – years**	69 (44–82)	68 (44-82)	72 (50-82)	0.16
**Body mass index (BMI) – kg/m^2**	27 (19-37)	26 (19-33)	27 (23-37)	0.83
**Smoking history – pack years**	10 (0–240)	10 (0-50)	30 (0 - 240)	**0.0006**
• **Current – n (%)**	7 (9)	5 (9)	2 (8)	0.36
• **Former – n (%)**	50 (64)	32 (59)	18 (75)	
• **Nonsmokers – n (%)**	21 (27)	17 (32)	4 (17)	
**Radiological diagnosis – n (%)**	40 (51)	23 (43)	17 (71)	**0.02**
**Histological diagnosis – n (%)**	38 (49)	31 (57)	7 (29)	
**FVC at diagnosis – L**	2.66 (1.53–4.61)	2.79 (1.67-4.36)	2.40 (1.53-4.61)	**0.03**
**FVC at diagnosis – %pred.**	77 (47–126)	79 (56–126)	72 (47–118)	0.08
**TLC at diagnosis - %pred.**	73 (40-96)	73 (45-96)	73 (40-93)	0.32
**DL_{CO} at diagnosis – %pred.**	56 (7–93)	56 (7-89)	56 (28-93)	0.60
**Gastroesophageal reflux – n (%)**	31 (40)	22 (41)	9 (38)	0.78
**Cardiovascular diseases – n (%)**	53 (68)	37 (69)	16 (67)	0.87
**Metabolic syndrome – n (%)**	33 (42)	22 (41)	11 (46)	0.67
**Pirfenidone– n (%)**	42 (54)	31 (57)	11 (46)	0.34
**Nintedanib – n (%)**	36 (46)	23 (43)	13 (54)	
**FVC decline in the 1st year– mL**	46 (-573–657)	59 (-573-657)	34 (-559-461)	0.70
**FVC decline in the 1st year – %pred.**	0 (-29-21)	1 (-29-21)	0 (-12-16)	0.80
**Stable – n (%)**	62 (79)	43 (80)	19 (79)	0.96
**Progressors – n (%)**	16 (21)	11 (20)	5 (21)	
**Follow up time - months**	51 (1 - 173)	55 (11 - 173)	42 (1 - 89)	0.05
**Transplanted – n (%)**	5 (6)	4 (7)	1 (4)	0.99
**Deaths – n (%)**	37 (47)	22 (41)	15 (63)	0.07

Values are expressed as numbers and (%) or median and ranges as appropriate. Negative values mean improvement of FVC. To compare demographic data and baseline clinical characteristics between TT / GT genotype and GG genotype, Chi square test and Fisher t test (n < 5) for categorical variables and Mann-Whitney U test for continuous variables were used. Values are expressed as numbers and (%) or median and ranges as appropriate. Negative values mean improvement of FVC. To compare demographic data and baseline clinical characteristics between TT / GT genotype and GG genotype, Chi square test and Fisher t test (n < 5) for categorical variables and Mann-Whitney U test for continuous variables were used.

Table S2. MUC5B rs35705950 genotype frequency.

		Observed	Expected	p Value
**T allele: 66/156 (42%)**	**TT genotype – n (%)**	**12 (15)**	**16 (17)**	
	**TG genotype – n (%)**	**42 (54)**	**43 (49)**	**0.82**
**G allele: 90/156 (58%)**	**GG genotype – n (%)**	**24 (31)**	**30 (34)**	

Chi square test for categorical variables was used.

Table S3. Radiological scores at treatment initiation (HRCT1) of the entire study population, and categorized in TT/TG genotype or GG genotype.

	**Entire Population (n =78)**	**TT/TG genotype (n =54)**	**GG genotype (n = 24)**	**p Value**
**Alveolar score - %**	20 (0-62)	22 (0-62)	16 (0-44)	0.53
**Honeycombing score - %**	2 (0-70)	2 (0-41)	3 (0-70)	0.54
**Interstitial score - %**	23 (0-52)	22 (0-52)	25 (0-45)	0.91
**Pooled interstitial score and honeycombing - %**	28 (8-89)	28 (9-73)	30 (8-89)	0.76

Values are expressed as median and ranges. To compare the radiological scores in HRCT1 between TT/GT and GG genotype groups, and Mann-Whitney U test for continuous variables was used.

Table S4. Radiological scores at treatment initiation (HRCT1) of the entire study population, and categorized in TT, TG genotype or GG genotype.

	**TT (n =12)**	**TG (n = 42)**	**p**	**GG (n = 24)**	**p (TT vs GG)**	**p (TG vsGG)**
**Alveolar score - %**	19 (0-35)	22 (0-62)	0.71	16 (0-44)	0.87	0.48
**Honeycombing score - %**	3 (0-28)	2 (0-41)	0.91	3 (0-70)	0.73	0.53
**Interstitial score - %**	23 (0-50)	22 (3-52)	0.76	25 (0-45)	0.78	0.80
**Pooled interstitial score and honeycombing - %**	28 (11-58)	28 (9-73)	0.88	30 (8-89)	0.99	0.71

Values are expressed as median and ranges. To compare the radiological scores in HRCT1 between TT / GT and GG genotype groups, and Mann-Whitney U test for continuous variables was used.

Table S5. Radiological scores at treatment initiation (HRCT1) and after one year of treatment (HRCT2) in TT,TG and GG populations.

**ALVEOLAR SCORE**			
	**HRCT1**	**HRCT2**	**p**
**TT**	19 (0 - 35)	23 (0 - 34)	0.18
**TG**	22 (0 - 62)	20 (0 - 64)	0.79
**GG**	16 (0 - 44)	18 (1 - 86)	**0.05**

**HONEYCOMBING SCORE**			
	**HRCT1**	**HRCT2**	**p**
**TT**	3 (0 - 28)	3 (0 - 31)	0.75
**TG**	1.5 (0 - 41)	7 (0 - 50)	**0.002**
**GG**	3 (0 - 70)	7 (0 - 83)	**0.007**

INTERSTITIAL SCORE			
	**HRCT1**	**HRCT2**	**p**
**TT**	23 (0 - 50)	23 (0 - 50)	0.50
**TG**	22 (3 - 52)	24 (3 - 59)	0.61
**GG**	26 (0 - 45)	26 (0 - 53)	0.15

pooled HONEYCOMBING AND INTERSTITIAL SCORE			
	**HRCT1**	**HRCT2**	**p**
**TT**	28 (11 - 58)	30 (16 - 60)	0.31
**TG**	28 (9 - 73)	31 (9 - 93)	**0.004**
**GG**	28 (8 - 89)	42 (8 - 98)	**0.002**

AS = alveolar score; IS = interstitial score; HC = honeycombing; HC+IS = pooled interstitial score and honeycombing. Values are expressed as median and range. P values refer to comparisons between HRCT1 and HRCT2, and Wilcoxon signed rank test for paired non parametric data was used.
